# MicroPulse Transscleral Laser Therapy Demonstrates Similar Efficacy with a Superior and More Favorable Safety Profile Compared to Continuous-Wave Transscleral Cyclophotocoagulation

**DOI:** 10.1155/2022/8566044

**Published:** 2022-02-08

**Authors:** Enrico Bernardi, Marc Töteberg-Harms

**Affiliations:** ^1^University of Zurich, Medical Faculty, Zurich, Switzerland; ^2^University Hospital Zurich, Department of Ophthalmology, Zurich, Switzerland; ^3^Department of Ophthalmology, Medical College of Georgia at Augusta University, Augusta, GA, USA

## Abstract

**Purpose:**

The aim of this study was to compare effectiveness and safety of MicroPulse transscleral laser therapy (MP-TLT) using the original MicroPulse P3® device and continuous-wave transscleral cyclophotocoagulation (CW-TSCPC) using the G-Probe® device in glaucoma.

**Methods:**

Spherical equivalent, intraocular pressure (IOP), best-corrected visual acuity (BCVA), and number of topical or oral ophthalmic pressure-reducing medications were registered at every time point, up to the last follow-up at 12 months. A complete slit-lamp examination was conducted to record the following complications: corneal edema, persistent ocular hypotony (IOP ≤5 mmHg) on two consecutive follow-up visits, choroidal detachment, phthisis bulbi, sympathetic ophthalmia, cystoid macular edema, or other abnormal ocular findings. Success was defined as IOP between 6 and 21 mmHg and >20% reduction in IOP with or without antiglaucoma medications.

**Results:**

47 eyes underwent MP-TLT and 150 CW-TSCPC. At 12 months, success was achieved in 88.6% in the CW-TSCPC group and 87.5% in the MP-TLT group (*p* = 0.883). In the CW-TSCPC group, eyes achieved a 42.4% IOP reduction (from 28.3 ± 12.3 mmHg to 15.3 ± 6.0 mmHg) and a 31.1% reduction (from 22.0 ± 7.2 mmHg to 15.7 ± 4.8 mmHg) in the MP-TLT group. Visual acuity remained primarily unaltered in both groups.

**Conclusion:**

MP-TLT was as effective in lowering IOP as CW-TSCPC and achieved comparable success. Additionally, MP-TLT demonstrated consistent and effective outcomes at every time point. The improved safety profile of MP-TLT allows the therapeutic spectrum to be broadened, granting ophthalmologists' treatment of glaucoma in earlier stages of glaucoma than those typically treated with CW-TSCPC, i.e., early to moderate and to patients with good central-vision.

## 1. Introduction

Glaucoma is a term describing a group of ocular disorders with a multifactorial etiology, but characterized by an intraocular, mostly pressure-associated, optic neuropathy [1–5]. As a chronic condition, it is among the leading causes of irreversible blindness worldwide [6–8]. The vision loss is caused by an irreversible damage to the optic nerve and a progressive loss of its nerve fibers [9–11]. However, an appropriate and timely therapy can effectively prevent nerve damage, loss of visual field, and hence blindness [12–15]. In most cases, glaucoma is associated with a pathological increase (>21 mmHg) of intraocular pressure (IOP). Therapies are aimed at reducing IOP to bring it within a normal and healthy range [16, 17].

There are several ways of achieving IOP reduction. Treatments fall within the categories of medications (mostly topical eye drops), laser procedures (i.e., laser trabeculoplasty, MicroPulse transscleral laser therapy (MP-TLT), or continuous-wave transscleral cyclophotocoagulation (CW-TSCPC)), or incisional surgeries (i.e., trabeculectomy, minimally invasive glaucoma surgery (MIGS), and endocyclophotocoagulation) [18–21].

CW-TSCPC is performed using the Cyclo G6® Laser System and the G-Probe® Delivery Device (IRIDEX Corp., Mountain View, CA). CW-TSCPC is an established cycloablative treatment that achieves IOP reduction through the destruction of the ciliary body to suppress aqueous humor production [22–26] using 810 nm laser energy [27, 28]. The footplate of the G-Probe is held parallel to the visual axis with the shorter edge of the footplate firmly between the anterior border and the middle of the limbus which places the laser fiberoptic over the pars plicata. The laser energy is absorbed by the melanin in the targeted pigmented epithelium of the ciliary body. Once photocoagulation is reached, a “pop” sound can be heard, indicating to the operator to reduce the power. Power is reduced in increments of 100 mW until no pop is heard before proceeding further with the treatment [29]. Although effective, this modality delivers an excessive amount of energy to the surrounding tissue, causing a high degree of collateral damage. Therefore, the use of CW-TSCPC is limited to refractory glaucoma due to the severe complications it can cause, such as persistent hypotony, persistent intraocular inflammation, hyphema, decreased visual acuity (VA), or phthisis bulbi [30].

To improve the safety profile of CW-TSCPC, IRIDEX developed the MicroPulse P3® Delivery Device. The MicroPulse P3 is a single-use, fiberoptic handpiece, [31, 32] compatible with the Cyclo G6® Laser, and is used to deliver MP-TLT. MicroPulse® technology (IRIDEX Corporation, Mountain View, CA, USA) delivers energy in a series of repetitive, short pulses of laser energy separated by longer rest periods to allow target tissue to gradually cool to avoid a cyclodestructive threshold. This “cooling” period also avoids collateral tissue damage, [33–42] which has been demonstrated histologically [43–45]. During MP-TLT, the laser energy is targeted to the pars plana rather than the pars plicata as it is in CW-TSCPC. The exact mechanism of action for IOP reduction is still uncertain, but is hypothesized that it is a combination of both increased trabecular and uveoscleral outflow in addition to aqueous suppression. The improved safety profile of MP-TLT over CW-TSCPC allows the therapeutic spectrum of treatment to be broadened, granting ophthalmologists a new approach to tackle glaucoma in earlier stages than those typically treated with CW-TSCPC [46–48].

The aim of this study is to assess the effectiveness and safety (i.e., change in VA after the procedure compared to baseline) of MP-TLT using the original MicroPulse P3 delivery device, compared with CW-TSCPC using the G-Probe delivery device. We hypothesize that reduction in IOP and medication will not differ significantly between both procedures.

## 2. Materials and Methods

A retrospective, comparative interventional study was conducted on patients with various types of glaucoma treated with CW-TSCPC or MP-TLT at the University Hospital Zurich (USZ), Zurich, Switzerland, between March 2016 and January 2020. The cantonal ethics commission of Zurich (KEK ZH) granted its approval to the study protocol, and the study follows the principles of the Declaration of Helsinki. As stated in the protocol and according to the Art. 34 HFG, the patients considered for this study signed either a general consent for research before the operation or an adapted consent specific for this study. The choice between CW-TSCPC and MP-TLT was left to the discretion of the surgeon, who based the choice on the type of glaucoma, the progression of the disease, the risk of intraoperative and postoperative complications of the surgical management, and most importantly, the patient's preference.

### 2.1. Inclusion and Exclusion Criteria

Patients included in the study had either a form of primary or secondary glaucoma and were diagnosed with moderate to advanced glaucoma. Patients excluded from the study were underage at the time of the operation or did not provide their agreement with either the general consent or the study-specific consent.

### 2.2. Procedure, Anesthesia, and Postoperative Care

All procedures were performed by one glaucoma specialist (T.H.M.). Before the session, all patients received either topical, peribulbar, or retrobulbar anesthesia. The topical option consisted of the “Topic Plus” method, which is a combination of topical unpreserved tetracaine 1% drops and lidocaine 2% nonalcoholic gel, and was reserved for patients undergoing MP-TLT. The peri- and retrobulbar anesthesia consisted of a 5 ml, 1 : 1 mixture of mepivacaine 2.0%, and carbostesine 0.5% plus hyalase 20 : 1. Intravenous sedation and analgesia was performed by 50 mg fentanyl plus thiopental sodium 0.5 g/20 ml, which was adapted to the patient's weight. Either the G-Probe or the MicroPulse P3 handpiece was used with the IRIDEX Cyclo G6 laser.

The CW-TSCPC treatment protocol utilized a laser power of 2,000 mW for a duration of 2,500 ms per spot. Fifteen laser spots were applied, sparing the superior aspect of the globe from 10 to 2 o'clock to preserve anatomy for future glaucoma interventions. 2% methylcellulose (Methocel, OmniVision, Puchheim, Germany) was used to guarantee a liquid interface.

MP-TLT was delivered using 2,000 mW of power at a duty cycle of 31.3%. 2% lidocaine gel or 2% methylcellulose was used as a coupling agent. The footplate of the MicroPulse P3 was placed at the limbus with its “notch” oriented toward the central cornea, which positioned the fiberoptic over the pars plana. The MicroPulse P3 probe was held perpendicular to the globe while applying steady pressure in a continuous sliding arc (sweeping) motion along the limbus for 80 seconds in the superior hemicircumference and then for an additional 80 seconds in the inferior hemicircumference, delivering approximately eight 10-second sweeps per 80 seconds.

For both, CW-TSCPC and MP-TLT, care was taken to avoid the 3 o'clock and 9 o'clock meridians, areas of scleral thinning, sites of filtering blebs, and glaucoma drainage devices. The eye was patched for 24 hours with a fixed combination ointment of tobramycin 3 mg/ml plus dexamethasone 1 mg/ml (Tobradex ointment; Alcon, Fort Worth, TX, USA). Thereafter, patients were started on topical fixed-combination tobramycin 3 mg/ml plus dexamethasone 1 mg/ml (Tobradex eye drops; Alcon, Fort Worth, TX, USA) 5x/d for 1 to 2 weeks. Patients were directed to continue with their preoperative antiglaucoma medication regimen, unless instructed otherwise. Medical hypotensive treatment was adjusted for each patient on every visit; it was reduced, when possible, in a stepwise approach and at the surgeon's discretion.

### 2.3. Baseline and Follow-Up Data Collection

To evaluate and compare CW-TSCPC and MP-TLT procedures, data gathered included preoperative age, sex, glaucoma type, number of glaucoma medications (drops and tablets), and IOP in millimeters of mercury (obtained either through Goldmann applanation tonometry or rebound tonometry when the first was not possible; just 16 patients (8%) underwent a rebound tonometry measurement at baseline and 7 (4%) at the last follow-up). In addition, best-corrected visual acuity (BCVA) and spherical equivalent was recorded. The participants underwent follow-up visits at 1 day, 1 week, 1 month, 3 months, 6 months, 9 months, and 12 months postoperatively. At each appointment, the following factors were registered: spherical equivalent, IOP, BCVA, and number of topical or oral ophthalmic pressure-reducing medications (62 patients (31%) were on oral acetazolamide therapy at baseline and none at the last follow-up). Simultaneously, a complete slit-lamp examination was conducted to record the following complications: corneal edema, persistent ocular hypotony (IOP ≤5 mmHg) on 2 consecutive follow-up visits, choroidal detachment, phthisis bulbi, sympathetic ophthalmia, cystoid macular edema, or other abnormal ocular findings. A loss of vision of two or more lines in BCVA compared to baseline or a loss of light perception vision were also noted and considered as complications.

### 2.4. Definition of Success and Failure

Success was defined as either an IOP between 6 and 21 mmHg at the last visit or an IOP reduction of >20% compared to the baseline measurement. Patients needing retreatment or invasive surgeries were considered as treatment failures. A retreatment session was contemplated no sooner than 3 months after the initial treatment for patients who failed to respond on 2 consecutive follow-ups. The decision to switch to a penetrating glaucoma surgery was left to the surgeon's discretion on a case-by-case basis.

### 2.5. Statistical Analyses

Excel 2016 was used for data management, and IBM SPSS Statistics (International Business Machines Corporation (IBM), Armonk, NY, USA) version 26 was used for statistical analysis. Descriptive statistics were reported as mean ± SD for continuous variables and as absolute values and percentage for categorical variables. Preoperative and postoperative data were compared using Student's *t*-test for equality of means (continuous variables) and chi-square test (categorical variables). A *p* value of <0.05 was considered to be statistically significant. Additionally, Kaplan–Meier survival analysis was computed for success rates using log rank Mantel-Cox test for differences between groups.

## 3. Results

In total, 197 eyes were treated with either CW-TSCPC (150 eyes) or MP-TLT (47 eyes). Primary open-angle glaucoma was the most prevalent (*n* = 69, 35.2%), followed by pseudoexfoliative glaucoma (*n* = 66, 33.7%) and angle-closure glaucoma (*n* = 13, 6.6%). There was no difference between groups regarding eyes, age, gender, spherical equivalent, BCVA, medications, and diagnosis (see Table 1). However, the baseline IOP was considerably lower among patients who underwent MP-TLT (22.0 ± 7.2 mmHg) compared to patients who received CW-TSCPC (28.3 ± 12.3 mmHg). Additionally, patients in the CW-TSCPC had more advanced visual field defects than those in the MP-TLT group (see Table 1).

### 3.1. CW-TSCPC

**Figure 1:**
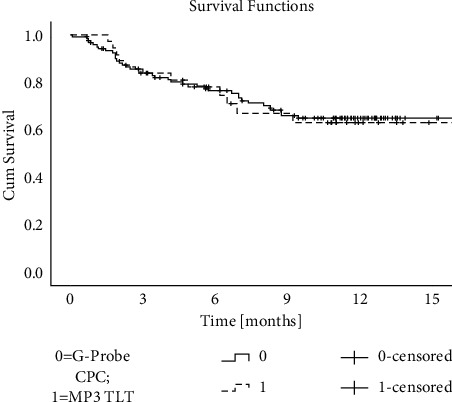
The Kaplan–Meier survival statistics (CPC = G-Probe cyclophotocoagulation, solid line; MP-TLT = MicroPulse laser treatment for glaucoma, dashed line) showed no difference between the success rates (*p* = 0.883).

The eyes treated with CW-TSCPC showed a 26.3% reduction in IOP (and a 13.4% reduction in meds) compared to baseline at day 1, 45.7% (16.7%) at 1 week, 35.7% (23.4%) at 1 month, 37.9% (26.7%) at 3 months, 44.5% (33%) at 6 months, 42.4% (30%) at 9 months, and 43.7% (33%) at 12 months. Differences at each follow-up time point were statistically significant (see Table 2). The success rate at 12 months was 88.6% (see Figure 1).

### 3.2. MP-TLT

MP-TLT achieved an IOP reduction of 5.6% (and a 7.14% reduction in meds) at day 1, 31.9% (10.7%) at 1 week, 24.7% (7.14%) at 1 month, 24.3% (3.57%) at 3 months, 25.0% (21.4%) at 6 months, 31.0% (10.7%) at 9 months, and 31.1% (7.14%) at 12 months (*p* values are shown in Table 2). The success rate at 12 months was 87.5% (see Figure 1).

### 3.3. Differences between CW-TSCPC and MP-TLT

The most notable differences between the two therapies are the rate at which IOP decreased, reduction of medications, and vision loss. MP-TLT reduced the IOP more gradually and consistently than CW-TSCPC. The number of antihypotensive medications was comparable at baseline (2.8 ± 1.4 in the MP-TLT group and 3.0 ± 1.4 in the CW-TSCPC group); however, the number of medications had a greater decrease in the CW-TSCPC (33% reduction) compared to the MP-TLT group (7.14% reduction). BCVA remained primarily unaltered in both groups (see Table 2). Success rates were similar in both groups: 88.6% in the CW-TSCPC group vs. 87.5% in the MP-TLT group (*p* = 0.883; see Figure 1).

## 4. Discussion

Based on the findings of this study, MP-TLT demonstrated similar effectiveness as CW-TSCPC after 12 months of follow-up. Both procedures achieved a significant decrease in IOP, and the number of medications was reduced compared to baseline at all visits. Strikingly, there was no loss of vision after MP-TLT; however, there was vision loss after CW-TSCPC. The success rates of CW-TSCPC and MP-TLT in this study were very satisfactory, with 88.6% and 87.5%, respectively.

This study found a slightly higher MP-TLT success rate than those reported by Williams et al. (67%) [41], Aquino et al. (75%) [31], and Nguyen et al. (78.6%) [36]. Williams et al. included mostly patients with refractory glaucoma, which could have proven nonresponsive to previous treatment options, which could explain the lower success rate compared to the other articles. Their baseline IOP was higher as well (31.9 ± 10.2 mmHg compared to 22.0 ± 7.2 mmHg in this study); thus, a lower final IOP could have been expected [41]. In the study by Aquino et al. more than half of the patients (58%) suffered from neovascular glaucoma (NVG). Efficacy and success in treating NVG with any surgical intervention are generally poor, which can explain the lower success in IOP control in their study [31]. Nguyen et al. treated patients with similar characteristics as those treated in our study, and they found a comparable IOP reduction. However, the success rate was slightly lower (78.6% vs. 87.5%), but the study had a much higher decrease in medications (from 3.0 ± 1.1 at baseline to 1.4 ± 1.0 at 12 months), a 53.4% decrease, compared to 7.14% in our study [36].

BCVA remained primarily unaltered in the MP-TLT group, whereas it decreased in the CW-TSCPC group. A decrease of BCVA could be explained by further glaucoma progression. Unfortunately, no visual field data in this retrospective chart-review was available to prove or rule out this hypothesis. However, the baseline BCVA in the CW-TSCPC group was worse in the CW-TSCPC group (2.0 logMAR in the CW-TSCPC vs. 1.4 in the MP-TLT group). The lower baseline BCVA could be a sign for more advanced glaucoma cases in this group.

CW-TSCPC has been studied and evaluated more extensively than MP-TLT, given the longer time it has been available to clinicians. This study reported a CW-TSCPC success rate at 12 months of 88.6%. This is a more favorable outcome than reported by Schlote et al. (74.2%), [49] Quigley (72%), [50] and Grueb et al. (40.0%) [51]. Schlote et al. had a majority (73.3%) of patients who had been previously operated for glaucoma, and the most prominent diagnosis (21.5%) was inflammatory glaucoma; these two factors could explain the slightly lower success rate of CW-TSCPC [49]. In the study by Quigley, most eyes had severe glaucoma, with 75% having BCVA <20/200. However, the study focused on different success rates for different laser parameters [50]. Grueb et al. had the lowest success rate reported, attributed to the high prevalence (26.7%) of CW-TSCPC as a primary surgical treatment and a different definition of success (IOP reduction ≥20% or 4 ≤ IOP ≤ 18 mmHg) [51].

In the present study, MP-TLT proved superior success rates to those achieved by Khodeiry et al. in their recently published study on slow-coagulation CW-TSCP (i.e., 60.6% at 1 year) [52]. The slow-coagulation protocol uses a power of 1,250 mW and a duration of 4,000 ms rather than 2,500 mW and 2,000 ms. This technique shows a higher safety profile than standard CW-TSCPC, with a low degree of postoperatory complications, despite the suboptimal success rate. It is, nevertheless, a noncomparative retrospective study with a moderate sample size.

MP-TLT demonstrated much better results than the recently introduced “Cyclo Mix” technique, performed using the Supra 810 nm SubLiminal® laser (Quantel Medical, Cournon d'Auvergne, France). A study by Waldo et al. reported an absolute success rate of only 30.4% and a relative one of 87% [53]. The study presented, however, a limited number of participants (23 eyes from 13 patients) and comes with the disadvantages regarding safety due to CW-TSCPC compared to MP-TLT alone. The study by Awoyesuku et al. obtained a higher IOP reduction at 6 months (38.2%) [54]. However, the study had a smaller sample size (13 eyes) and had a higher baseline IOP (27.4 mmHg) [54]. Magacho et al. found slightly lower IOP after double-session MP-TLT. However, during double-session MP-TLT, each hemisphere was treated for approximately 358 to 363 sec [55]. The longer duration of treatment and the higher energy resulted in more complications, e.g., 13 out of 185 cases had persistent mydriasis [55].

Finally, MP-TLT demonstrated equivalency in IOP reduction (31.1%) compared to “controlled cyclophotocoagulation” (COCO) (34%) at 12 months of follow-up based on benchmark study results by Lenzhofer et al. [56]. COCO incorporates a complex and costly optical feedback mechanism that automatically adjusts the applied laser energy during laser delivery [57, 58]. Stunningly, the much simpler and less costly MP-TLT techniques showed no inferiority compared to COCO.

VA was not negatively impacted by MP-TLT, which is corroborated by the literature [34, 59]. Thus, MP-TLT can be offered as a treatment option to patients not only with refractory glaucoma but also with early to moderate glaucoma and patients with good central vision.

Due to the retrospective nature of this study, a detailed analysis on complications was not conducted, as data in medical charts on this topic are usually inconsistent. As a surrogate for safety, we found little to no change in BCVA after MP-TLT and noticed an improvement after CW-TSCPC. The main focus was on efficacy of the techniques, given that the safety profile has already been acknowledged in other studies [31, 48]. The lack of adverse events in the MP-TLT group has been attributed to the fragmentation of laser delivery and overall lower energy applied to the tissues [60, 61]. The relevance of this study is highlighted by the absolute lack of evidence comparing MP-TLT and CW-TSCPC, as well as the relative lack of data on long-term outcomes, i.e., studies with a follow-up of more than two years [48, 62]. The study by Aquino et al. attained a follow-up of 78 months, where 67% of the 14 remaining patients had a 39% (range 31–68%) reduction in IOP from baseline [63].

The limitations of this study are related to the categorization of our hospital as a tertiary and quaternary care center. Many patients return to their private ophthalmologists for the postoperative follow-up visits, and their data become more difficult to obtain, hence the gaps in our data gathering and statistical analysis. This was furthermore unaided by the retrospective nature of this study.

In conclusion, MP-TLT proved to be a reliable and effective IOP-lowering technique and consistently lowered and maintained IOP throughout the 12 months of follow-up. Furthermore, medications were reduced compared to baseline at all time points. Overall, the effectiveness of MP-TLT is comparable to CW-TSCPC, despite utilizing less energy, hence causing less collateral tissue damage, pain, and inflammation. Strikingly, no loss of vision was found after MP-TLT, which enables ophthalmologists to consider MP-TLT as a valid therapeutic option even before fistulating surgery in patients with not only advanced and refractive glaucoma but also in patients with early and moderate glaucoma and good central vision.

## Figures and Tables

**Table 1:** Demographical data.

	All	G-Probe CPC	MicroPulse-TLT	*p* value
Eyes	197	150	47	1.000
	OD 98 (49.7%)	OD 75 (50.0%)	OD 23 (48.9%)	
	OS 99 (50.3%)	OS 75 (50.0%)	OS 24 (51.1%)	
Mean age (years)	72.1 ± 15.1 years	73.1 ± 15.5	68.9 ± 13.3	0.072
Gender	90 males (45.7%), 107 females (54.3%)	68 males (45.3%), 82 females (54.7%)	22 males (46.8%), 25 females (53.2%)	0.868
Spherical equivalent	−1.74 ± 4.79	−1.5 ± 4.6	−2.7 ± 5.3	0.320
Baseline BCVA (logMAR)	1.8 ± 2.4	2.0 ± 2.5	1.4 ± 2.2	0.137
Baseline IOP (mmHg)	26.5 ± 11.6	28.0 ± 12.3	21.7 ± 7.2	<0.001
Baseline AGD	3.0 ± 1.4	3.0 ± 1.4	2.9 ± 1.4	0.501
Baseline VF MD (dB)	15.4 ± 9.1	18.3 ± 18.2	8.1 ± 6.4	0.003
Diagnosis				
Primary open-angle glaucoma	69 (35.2%)	48 (32.0%)	21 (45.7%)	0.460
Pseudoexfoliative glaucoma	66 (33.7%)	55 (36.7%)	11 (23.9%)	
Pigment dispersion glaucoma	5 (2.6%)	3 (2.0%)	2 (4.3%)	
Angle-closure glaucoma	13 (6.6%)	9 (6.0%)	4 (8.7%)	
Aphakic glaucoma	10 (5.1%)	8 (5.3%)	2 (4.3%)	
Ocular hypertension	2 (1.0%)	2 (1.3%)	0 (0%)	
Other glaucomas	31 (15.8%)	25 (16.7%)	6 (13%)	

OD = right eye, OS = left eye, BCVA = best-corrected visual acuity, logMAR = logarithm of the minimum angle of resolution, IOP = intraocular pressure, mmHg = millimeters of mercury, VF MD = visual field mean defect, dB = decibel.

**Table 2:** Preoperative and postoperative data for BCVA, IOP, and Meds.

		Baseline	1 day	*p*	1 week	*p*	1 month	*p*	3 months	*p*	6 months	*p*	9 months	*p*	12 months	*p*
BCVA (logMAR)	G-probe CPC	2.0 ± 2.5	2.2 ± 2.5	0.051	2.6 ± 3.2	0.002	2.0 ± 2.5	0.052	1.8 ± 2.3	0.531	1.5 ± 2.2	0.537	2.3 ± 2.9	0.178	1.7 ± 2.4	0.308
	MicroPulse-TLT	1.4 ± 2.3	1.2 ± 1.9	0.994	1.8 ± 3.4	0.203	1.6 ± 2.9	0.459	1.6 ± 2.9	0.029	1.5 ± 2.8	0.058	1.6 ± 3.0	0.058	1.5 ± 2.8	0.525
	*p*	0.168	0.031	—	0.285	—	0.445	—	0.754	—	0.893	—	0.420	—	0.731	—

IOP (mmHg)	G-probe CPC	28.3 ± 12.3	20.8 ± 9.3	<0.001	15.1 ± 9.3	<0.001	18.1 ± 10.7	<0.001	17.1 ± 7.0	<0.001	16.0 ± 7.6	<0.001	15.6 ± 6.6	<0.001	15.3 ± 6.0	<0.001
	MicroPulse-TLT	22.0 ± 7.2	20.8 ± 7.7	0.122	15.2 ± 5.6	<0.001	16.7 ± 6.0	<0.001	16.3 ± 3.6	0.003	16.7 ± 4.3	0.001	17.1 ± 6.7	0.092	15.7 ± 4.8	0.005
	*p*	0.001	0.970	—	0.942	—	0.477	—	0.600	—	0.647	—	0.459	—	0.815	—

Meds	G-probe CPC	3.0 ± 1.4	2.6 ± 1.6	<0.001	2.5 ± 1.5	<0.001	2.3 ± 2.4	0.002	2.2 ± 1.3	<0.001	2.0 ± 1.4	<0.001	2.1 ± 1.5	0.001	2.0 ± 1.1	<0.001
	MicroPulse-TLT	2.8 ± 1.4	2.6 ± 1.5	0.032	2.5 ± 1.3	0.041	2.6 ± 1.3	0.115	2.7 ± 1.2	0.260	2.2 ± 1.3	0.027	2.5 ± 1.2	0.502	2.6 ± 1.2	0.551
	*p*	0.422	0.924	—	0.975	—	0.542	—	0.071	—	0.564	—	0.470	—	0.060	—

BCVA = best-corrected visual acuity, logMAR = logarithm of the minimum angle of resolution, IOP = intraocular pressure, mmHg = millimeters of mercury.

## Data Availability

The data used to support the findings of this study are restricted by national law in order to protect patient privacy. Data are available from the authors on request and after prior ethical approval to share data.
